# HPTLC screening of saccharin in beverages by densitometry quantification and SERS confirmation†

**DOI:** 10.1039/d1ra09416e

**Published:** 2022-03-16

**Authors:** Qifei Chen, Huaming Hou, Dan Zheng, Xueming Xu, Xingjun Xi, Yisheng Chen

**Affiliations:** School of Food Science and Technology, Jiangnan University Wuxi 214122 China chenyisheng@sxau.edu.cn; College of Food Science and Engineering, Shanxi Agricultural University Taigu Shanxi 030801 China; Sub-Institute of Agriculture and Food Standardization, China National Institute of Standardization Beijing 100191 China

## Abstract

As a widely used artificially synthesized sweetener, saccharin faced numerous disputes associated with food safety. Therefore, its fast analysis in food is of crucial importance. In this study, an analytical method for the fast and reliable screening of saccharin in various beverages was established and validated, by combining HPTLC with densitometry and surface enhanced Raman spectroscopy. The diluted sample liquid was directly sprayed and separated on a silica gel plate using a mixture of ethyl acetate and acetic acid in the ratio of 9 : 1 (v/v) as the mobile phase. The separation realized full isolation of the analyte from background noises. Then, a densitometry analysis in the absorption–reflection mode (working wavelength 230 nm) was optimized to obtain quantitative data, showing a good linearity in the range of 40–200 ng per band (*R*^2^ = 0.9988). The limits of detection and quantification were determined to be 6 and 20 ng per band, respectively, which were equal to 6 and 20 mg kg^−1^. The quantitative results also displayed satisfactory accuracy and precision, with a spike-recovery rate within 87.75–98.14% (RSD <5.13%). As a cost-efficient tool for confirmation, surface enhanced Raman spectroscopy was employed to profile the molecular fingerprint of the analyte eluted from the plate layer. Under optimized conditions (785 nm laser as the excitation light and silver nanoparticle loaded glass fiber paper as the active substrate), the elution of the saccharin band exhibited stable and sensitive surface enhanced Raman spectroscopy signals. This study demonstrated that HPTLC could be a versatile platform for food analysis, with outstanding simplicity and cost-efficiency.

## Abstract 2

As a widely used artificially synthesized sweetener, saccharin faced numerous disputes associated with food safety.

## Graphical abstract

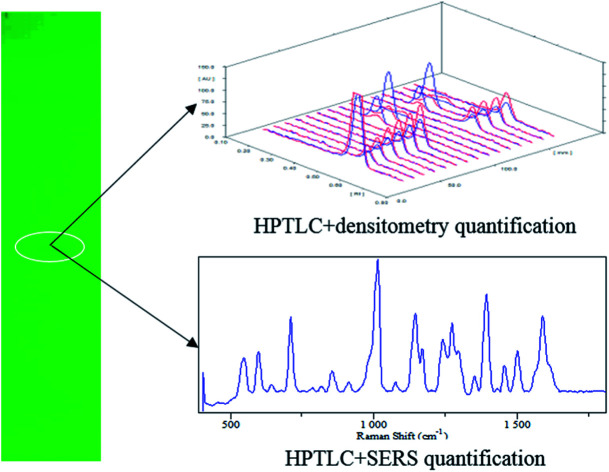

## 1 Introduction

Saccharin is the first artificially synthesized sweetener with a remarkably high sweetness. As an important additive in the food industry, the safety issue of saccharin, however, became disputable since the 1970s, concerning its potential adverse health effects.^1,2^ Moreover, exposure to saccharin may disrupt intestinal epithelial cell barrier function *in vitro*, as evidenced by Santos.^3^ As such, the Food and Drug Administration of the United States officially prohibited the addition of saccharin into food once in 1977, which was repealed later. Nevertheless, strict regulations on the usage of saccharin in food is stipulated around the world. According to the national standard GB2760-2014 in China, the allowable amount of saccharin used in food and beverages is 150–500 mg kg^−1^.^4^

Nevertheless, the abuse of saccharin is still frequently reported due to its high cost-efficiency. Therefore, it becomes particularly important for the accurate, fast and convenient detection of saccharin. The commonly used detection methods for saccharin are high performance liquid chromatography (HPLC),^5^ mass spectrometry (MS),^6^ combination of liquid chromatography and mass spectrometry (HPLC-MS),^2^ ion chromatography,^7^ capillary electrophoresis,^8^ and differential kinetic spectrophotometry.^9^ However, these methods faced some intrinsic problems, such as poor matrix tolerance and throughput, greatly hindering their usage in screening tasks.

In recent years, high performance thin layer chromatography (HPTLC) has become an important analytical tool because of its simplicity, high throughput, and cost-efficiency.^10–13^ It has been widely used in medicine, food, cosmetics and environmental analysis.^14–16^ As a flexible separation platform, HPTLC can realize unlimited compatibility with off-line detection technologies. At present, HPTLC is not only combined with densitometry or camera,^17,18^ but also effectively coupled to state-of-the-art detectors, such as MS,^19^ biosensors,^20^ and surface enhanced Raman spectroscopy (SERS).^21^

SERS can give fingerprint-like structural information of the targeted compounds. Besides, the spectrum strength can be abnormally enhanced *via* the electro-magnetic coupling with the active substrate, resulting in extremely high sensitivity even down to a single molecule.^22–25^ Nevertheless, SERS is vulnerable to food matrices, when used alone. More specifically, any compounds having higher affinity to the surface of the active substrate may lead to the suppression of the analyte signal in the mixture. This greatly limits the application of SERS in food analysis. In combination with HPTLC, this weak point can be easily overcome. In this way, the analyte is spatially isolated from the interfering matrices staying at the plate after separation, which can be easily coupled to SERS measurements.

As such, this study combined HPTLC-densitometry-SERS for the detection of saccharin. HPTLC was used as the separation platform, of which the result was directly quantified by densitometry. In order to enable sensitive SERS detection, a flexible substrate with high SERS activity was fabricated based on the hybrid of silver nanoparticles (AgNP) and glass fiber paper. The combination of HPTLC with SERS was performed in the off-line mode, giving sensitive and stable fingerprint information of the targeted band as conclusive confirmation.

## 2 Materials and methods

### 2.1 Chemicals and materials

The analytical standard of saccharin (>99.5%) was obtained from Aladdin (Shanghai, China). Methanol (≥99.8%), ethyl acetate (≥99.5%), acetic acid (≥99.5%), l-ascorbic acid (≥99.8%), sodium hydroxide (≥99.5%), sodium borohydride (≥96%), silver nitrate (≥99.8%), glass fiber membrane, aluminum foil and beakers were obtained from Sinopharm Chemical Reagent Co., Ltd. (Beijing, China). Class backed silica gel F_254_ plates (20 × 10 cm, analytical grade, serial No. 4022536067940) were supplied by Merck (Darmstadt, Germany). Prior to using, each plate was pre-washed with methanol, followed by drying at 120 °C for 20 min on a TLC Plate Heater (CAMAG, Muttenz, Switzerland). Four beverage samples (1-iced tea, 2-cola, 3-salt soda water, 4-alcoholic drink) were purchased at local supermarkets.

### 2.2 Standard solutions and samples preparation

The stock solution of saccharin was prepared by dissolving 10.0 mg saccharin in 10 mL methanol. Working solutions for assessing the dynamic range 40–1000 ng per zone were prepared by dilution to 0.2 and 0.02 mg mL; while for assessing the SERS activity of the fabricated substrate, the work solution was diluted to 0.1, 0.01, 0.001, 0.0001, and 0.00001 mg mL^−1^ with methanol. Principally based on the procedure used by Bruno,^13^ the 10 mL beverage liquid was first de-gassed under the ultrasonic bath for 10 min and then alkalized to pH = 7.0 and finally diluted 5 times with methanol. The liquid was purified by filtering through a 0.45 μm membrane, in case of needle blockage.

### 2.3 Flexible SERS substrate fabrication

The flexible substrate with high and stable SERS activity was fabricated by combining glass fiber paper and AgNPs, principally based on the *in situ* reduction method previously optimized in our laboratory.^26,27^ Briefly, four beakers were filled with a silver nitrate solution (0.02 mol L^−1^), ultra-pure water, sodium borohydride solution (0.02 mol L^−1^) and ultra-pure water, respectively, and the beakers were placed on a shaker at an oscillation frequency of 120 rpm. Then, a piece of glass fiber paper was soaked for 30 s in each of the four beakers successively. The first reduction step was completed by repeating this process 8 times. After that, the glass fiber paper was sequentially impregnated into four beakers filled with AgNO_3_ solution (0.02 mol L^−1^), ultra-pure water, l-ascorbic acid solution (0.02 mol L^−1^) and ultra-pure water for 60 s under shaking. The second reduction step was completed by repeating this process 4 times. Finally, the obtained material was rinsed with ultra-pure water 3 times to remove the reactants and stored in ultra-pure water. The fabricated SERS substrates were characterized by scanning electron microscopy.

### 2.4 HPTLC

Under a 0.4 MPa nitrogen stream, the liquid of beverage samples (5 μL) and saccharin standard solutions were sprayed as 6 mm bands onto a 20 × 10 cm silica gel plate, with a semiautomatic TLC sampler Linomate 5 (CAMAG, Muttenz, Switzerland) equipped with a 100 μL syringe. The application settings were 10 mm from the lower edge, 30 mm initially from the left side, and application speed 100 nL s^−1^. Then, the plate was developed with the automatic development chamber ADC-2 (CAMAG, Muttenz, Switzerland) using ethyl acetate + acetic acid 9 : 1 (v/v) as the mobile phase. In order to realize standardized and repeatable separation conditions, the following settings were used: 60 s pre-drying, 3 min humidity control (relative humidity 33%), 10 min tank saturation, 10 min plate pre-conditioning, and 80 mm migration distance. After separation, the plate was dried at 80 °C for 5 min on a TLC plate for the removal of organic residues. After chromatography, the image of the separation result was documented on a DD70 HPTLC imaging system (Biostep, Germany) equipped with a Sony EOS700D digital camera under 254 nm light illumination.

### 2.5 Densitometry quantification

To obtain quantitative data of separation, TLC Scanner 3 (CAMAG, Muttenz, Switzerland) was used to scan the plate in the absorption-reflection mode, with the following settings: slit dimensions 3.0 × 0.30 mm (Micro), optical system for maximum resolution, scanning speed 20 mm s^−1^, data resolution 100 mm per step, deuterium and tungsten lamp, and working wavelength 230 nm. Data acquisition and processing was done using the CAMAG winCATS software in quantitative mode version 1.4.8. The quantification was based on the peak area. From the calibration curve, the limit of detection (LOD) and limit of quantification (LOQ) were determined: 
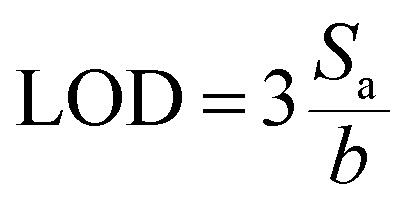
, 
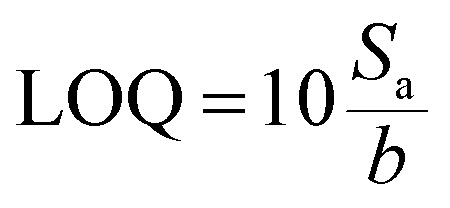
, where *S*_a_ is the standard deviation of the *y*-intercepts, of regression lines, *b* as the slope of the calibration curves of regression lines, according to the method of Shrivastava and Gupta.^28^

### 2.6 Off-line SERS analysis

Principally based on the procedure described by Chen,^29^ the Raman and SERS response of saccharin were recorded on a DXRxi laser confocal microscopy Raman spectrometer (Thermo Fisher, USA), with the following settings: incident laser wavelength 785 nm and signal acquisition averaged from three times accumulations of 10 s. To evaluate the activity of the substrate, pieces of the as-prepared substrate were immersed into the pure saccharin solutions (1, 0.1, 0.01, 0.001, 0.0001, 0.00001 mg mL^−1^) for 5 min. Then, they were placed on a piece of clean aluminum foil for SERS measurements. As for the saccharin separated on the silica gel plate, the SERS measurement was performed in the off-line mode. Briefly, bands visualized under 254 nm ultraviolet light were marked by a soft pencil and then scraped off with a pointed scraper. The obtained powder was collected and transferred to a vial filled with 0.1 mL methanol. After that, the mixture was conditioned in an ultrasonic bath for 10 min in order to elute the saccharin from the silica gel powder. Finally, the SERS response of the elution was recorded. The obtained data was processed with LabSpec 5 software, 9.10.27 version.

## 3 Results and discussion

### 3.1 HPTLC separation

To exclude potential interferences from co-extracted matrices, the mobile phase of separation was optimized first. From a trial test, the mixture of ethyl acetate and acetic acid 9 : 1 (v/v) was found to give the best separation result, in which the *R*_f_ of saccharin was 0.52. It was noteworthy to mention that the separation was not repeatable if the development step was performed manually. The retention of the analyte significantly varied between each run. Moreover, serious edge-effect can be observed without sufficient humidity control and plate condition. This laterally evidenced the necessity of instrumental operation for HPTLC analysis.

### 3.2 Densitometry quantitation and validation

**Fig. 1:**
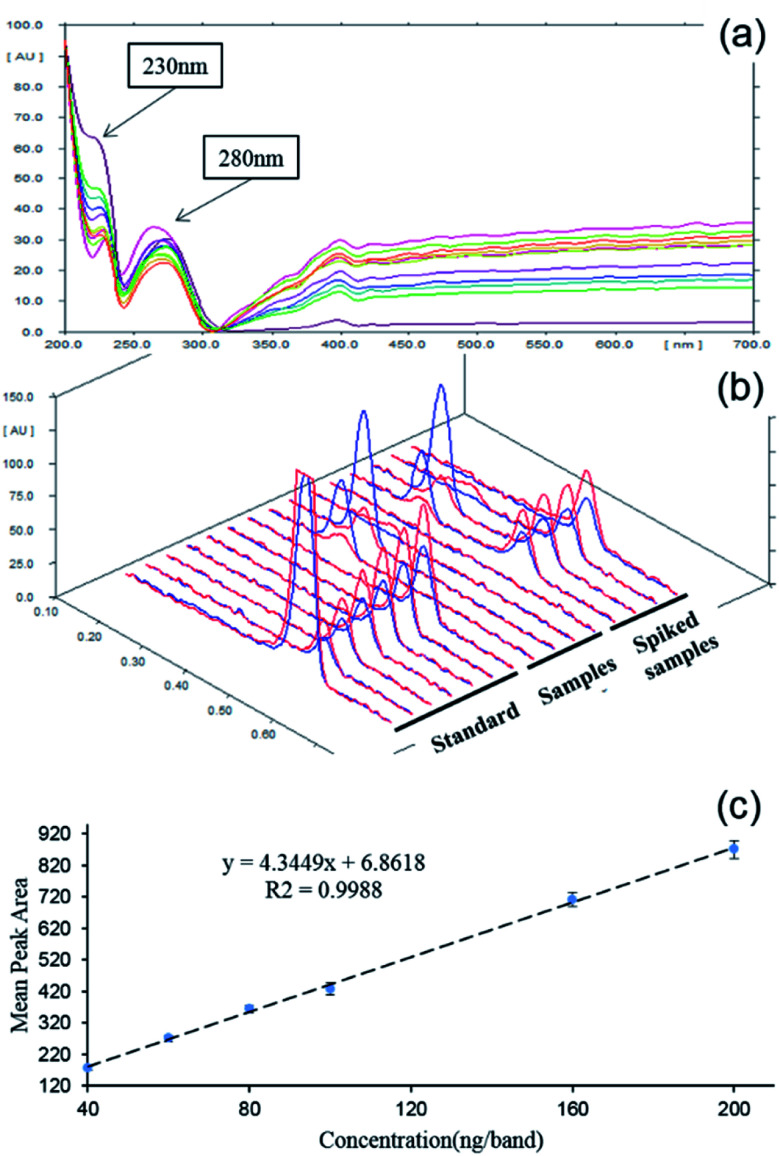
Absorption spectrum (200–700) of saccharin spotted on the HPTLC plate (a); densitogram obtained from different excitation wavelength: 230 nm-red line and 280 nm-blue line (b); the calibration curve of saccharin bands within 40–200 ng per band (c).

The absorption-reflection mode of densitometry was selected for scanning quantification. In order to locate the optimized work wavelength, the absorption spectrum of saccharin deposited on the HPTLC plate was profiled 200–700 nm. From Fig. 1a, it was clear that the spectrum displayed two absorption peaks at 230 nm and 280 nm, and the former was of relatively higher intensity. On this basis, the performance of the two candidate wavelengths was further estimated by a dual wavelength measurement. As comparatively shown in Fig. 1b, the saccharin peak was of better intensity when 230 nm was used as the incident wavelength. This also agreed well with previous reports.^10,12^ Therefore, 230 nm was chosen as the working wavelength. With optimized parameters, 6 bands of the saccharin standard with gradient concentrations (40, 60, 80, 100, 160, 200 ng per band) were quantified. From Fig. 1c, it could be observed that the densitometry results showed good linearity (*R*^2^ = 0.9988) within a broad range from 40 to 200 ng per band.

As shown in Table 1, the calculated LOD and LOQ of saccharin under this method were 6 ng per band and 20 ng per band, respectively. Similarly, based on the 5 μL sampling volume and 5 times dilution, it could be calculated that the LOD and LOQ were 6 mg kg^−1^ and 20 mg kg^−1^, respectively, which were completely sufficient to meet the detectability demanding for saccharin in the beverage (150 mg kg^−1^). To further evaluate the quantitative accuracy and precision of the HPTLC-densitometry method, the recovery rates of four spiked samples were analyzed. The real sample was spiked at three different levels: 50 ng per band, 100 ng per band and 150 ng per band. As summarized in Table 2, the average recovery rates of spiked samples 1–4 were 93.35%, 92.90%, 94.32% and 89.97%, respectively. The recovery rates of spiked samples were in the range of 87.75–98.14%, which showed that the established HPTLC-densitometry detection was of good accuracy.

**Table 1:** The quantitative performance of the optimized HPTLC-densitometry

Parameters	Saccharin
*R*_f_	0.52
Linearity range (ng per band)	40–200
Correlation coefficient (*R*^2^)	0.9988
Standard deviation of the *y*-intercepts (*S*_a_)	8.70
Slope of the calibration curves (*b*)	4.34
LOD^a^ (ng per band)	6
LOQ^a^ (ng per band)	20

a LOD and LOQ were calculated according to the method of Alankar Shrivastava.

**Table 2:** Assessment of the accuracy and precision of HPTLC-densitometry quantification

Samples	Spiked (ng per band)	Detected (ng per band)	Recovery^a^ (%)	RSD^a^	Average Recovery (%)
Sample 1	50	49.07 ± 1.11	98.14 ± 2.22	2.19	93.35
	100	89.79 ± 3.10	89.79 ± 3.10	3.39	
	150	138.17 ± 3.65	92.11 ± 2.43	2.61	
Sample 2	50	45.76 ± 2.43	91.53 ± 4.86	5.13	92.90
	100	94.57 ± 1.87	94.57 ± 1.87	1.95	
	150	138.90 ± 3.88	92.60 ± 2.59	2.76	
Sample 3	50	45.82 ± 1.35	91.65 ± 2.69	2.84	94.32
	100	94.55 ± 2.42	94.55 ± 2.42	2.52	
	150	145.14 ± 3.79	96.76 ± 2.53	2.58	
Sample 4	50	45.55 ± 1.50	91.10 ± 3.00	3.19	89.97
	100	87.75 ± 1.50	87.75 ± 1.50	1.68	
	150	136.59 ± 2.52	91.06 ± 1.68	1.82	

a The value was the average of 3 parallel measurements.

As mentioned before, one of the major purposes of this study was to achieve the fast quantification of saccharin in different beverage samples. In order to further evaluate its real applicability, the key performances (detectability, accuracy, efficiency and reliability) of the proposed method were compared with those of the relevant methods in the literature,^13,30,31^ as tabulated in Table S1 (see the ESI†). It was apparent that the method developed in this study displayed a remarkably better balance between different aspects.

### 3.3 SERS substrate fabrication

**Fig. 2:**
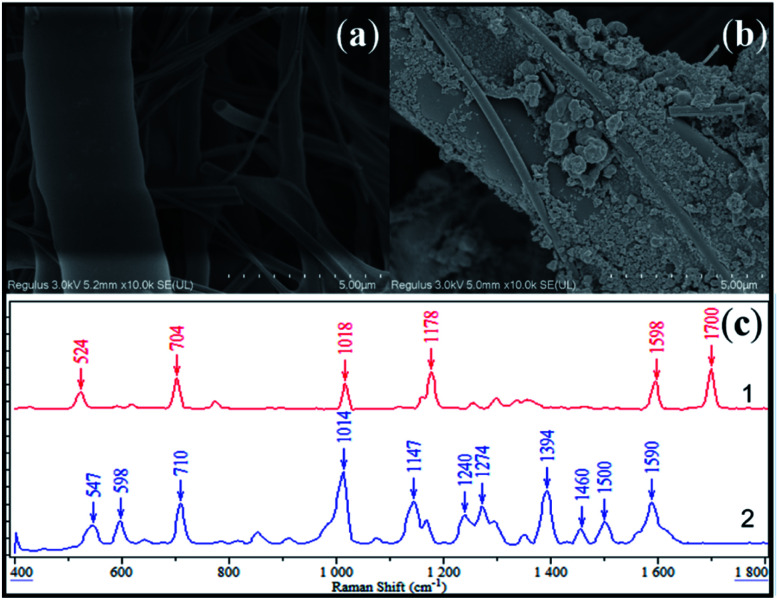
SEM image of blank glass fiber filter (a), SEM image of glass fiber filter loaded with AgNPs (b), Raman scattering responses of saccharin in the form of 1-standard solid and 2–1 mg mL^−1^ methanol solution (c).

The fabricated SERS active substrate was characterized *via* scanning electron microscopy (SEM). From Fig. 2a and b, it could be observed that the as-synthesized AgNPs attached onto the surface of the glass fiber, resulting in the stabilization of SERS activity. Fig. 2c compared the SERS response of saccharin with the as-prepared substrate with normal Raman spectra of saccharin powder. It was apparent that both results displayed high similarity to each other, sharing main peaks at 710, 1014, 1147 and 1590 cm^−1^. By referring to previous publications,^32–34^ it was further evidenced that these characteristic peaks could be attributed to the SERS fingerprint of saccharin, as shown in Table 3.

**Table 3:** The main SERS characteristic peaks and their attributions of the saccharin solution

Peak position (cm^−1^)		Vibrational Modes
Saccharin SERS	Reference	
710	709	C_6_ ring stretching
705		
704		
1018	1016	
1014		
1146	1145	C_5_–H_16_ & C_6_–H_17_ rocking
1147		
1274	1292	N_9_–C_7_ stretching
1296		
1590	1586	C=C stretching

**Fig. 3:**
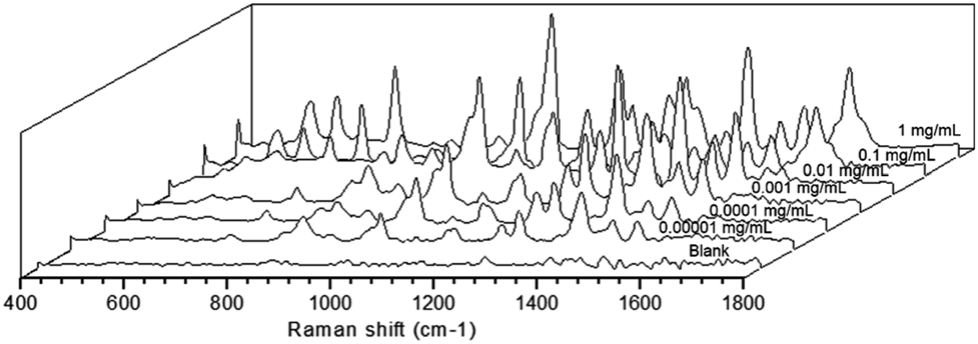
Sensitivity assessment of the as-prepared SERS substrate to the working solution of saccharin.

Apart from that, the SERS activity of the as-prepared substrate was further estimated by analyzing the working solution of saccharin at different concentrations. As shown in Fig. 3, clear fingerprint peaks of the analyte could still be observed, even when the work solution was diluted to 0.00001 mg mL^−1^. This evidenced that the as-prepared substrate was sensitive enough.

### 3.4 SERS confirmation of HPTLC results

**Fig. 4:**
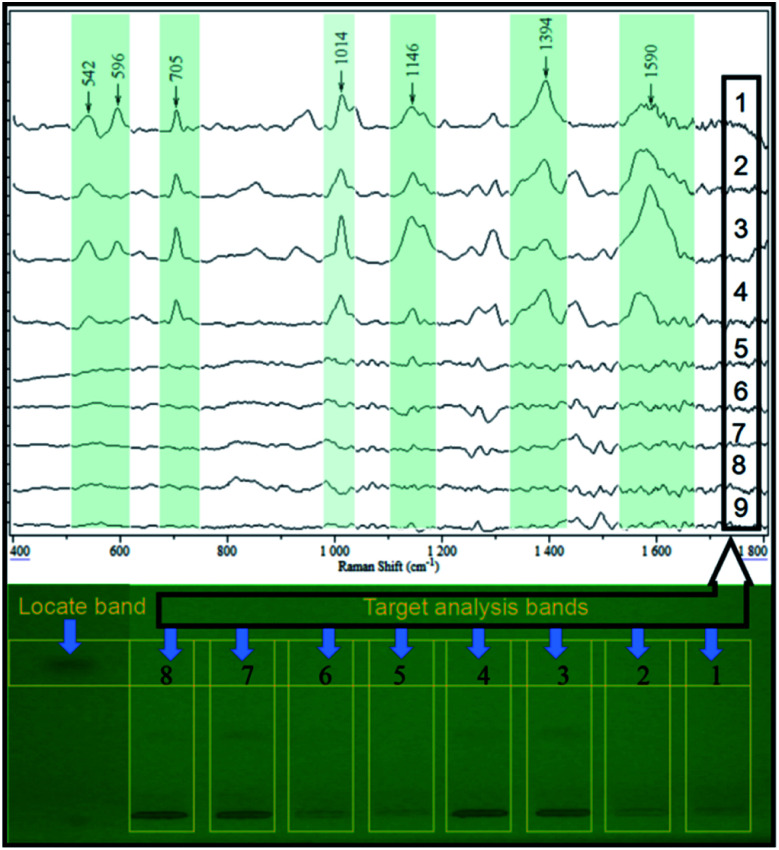
Result of SERS qualitative analysis on the four real samples and four 50 ng per band spiked samples (1–4 for spiked samples 1–4, 5–8 for real samples 1–4, 9 for the blank control silica gel plate).

A different conventional *in situ* linking, the combination of HPTLC and SERS, was performed in the off-line mode in this study. This was out of two considerations. First, the direct deposition of AgNPs onto the surface of the HPTLC plate may lead to the variation or even inactivation of its SERS activity, resulting in poor repeatability and detectability. On the contrary, the SERS activity of AgNPs can be stabilized when attached to the glass fiber. Therefore, the band of interest was scraped, of which the elution was a jointed assay with the as-prepared flexible substrate. In order to assess the real application, the elution of the sample band on the silica gel plate was analyzed by SERS. As shown in Fig. 4, the characteristic peaks of the spiked samples (50 ng per band) were similar to the SERS fingerprint of the saccharin standard, while the elution of the blank sample displayed insignificant SERS response. This could be used as an irrefutable evidence to eliminate false-positive results due to sample matrices. More importantly, the SERS signal of the analyte still displayed high intensity at the LOD of densitometry, ensuring the practical detection of saccharin.

## 4 Conclusions

In this study, HPTLC was used as an analytical platform, combining densitometry quantification and SERS confirmation, for the cost-efficient and reliable screening of saccharin in beverages. Under optimized conditions, the quantification of HPTLC-densitometry achieved a good linear relationship (*R*^2^ = 0.9988) and precision (RSD <5.13%). The combination of HPTLC and SERS detection further strengthened the reliability of the method, achieving the unambiguous identification of the analyte at the molecular level. With the as-prepared flexible substrate, the SERS sensitivity of saccharin could reach 0.00001 mg mL^−1^. The elution of the saccharin band exhibited stable and sensitive SERS signals. Compared with other commonly used detection methods, this method displayed remarkable advantages in high simplicity, throughput and matrix-tolerance.

## Author contributions

Qifei Chen: resources, validation; Huaming Hou: writing-revised draft; Dan Zheng: writing-revised draft; Xingjun Xi: writing-revised draft, conceptualization: Xueming Xu: conceptualization; Yisheng Chen: funding acquisition, conceptualization, writing –original draft/revised draft.

## Conflicts of interest

There are no conflicts of interest to declare.

## Supplementary Material

RA-012-D1RA09416E-s001
