# Supplementary material for: HPTLC screening of saccharin in beverages by densitometry quantification and SERS confirmation
Source: RSC Adv. 2022 Mar 16;12(14):8317–22. doi: 10.1039/d1ra09416e (PMC8984960; doi:10.1039/d1ra09416e)
Supplement: RA-012-D1RA09416E-s001 [file RA-012-D1RA09416E-s001.pdf]

Electronic Supplementary Material (ESI) for RSC Advances.
This journal is © The Royal Society of Chemistry 2022

Supplementary data

HPTLC screening of saccharin in beverage by densitometry quantification and SERS confirmation

Qifei Chen[a,b], Huaming Hou[b], Dan Zheng[b], Xueming Xu[a], Xingjun Xi*[c], Yisheng Chen[a,b]*

[a] School of Food Science and Technology, Jiangnan University, Wuxi 214122, China
[b] College of Food Science and Engineering, Shanxi Agricultural University, Taigu, Shanxi 030801, China
[c] Sub-Institute of Agriculture and Food Standardization, China National Institute of Standardization, Beijing 100191, China

*Corresponding author: Yisheng Chen, Xingjun Xi
E-mail address: chenyisheng@sxau.edu.cn

Table S1 The comparison of detection performance between the previously reported method and the proposed method in this work.

Analytical tools	LOD (mg/kg)	Spike-recovery (%)	Efficiency (min/sample)	Molecular fingerprint	Reference
HPTLC-FLD-SERS	6.0	87.55-98.14	2<*	Yes	This work
HPLC-Fluorescence	4.4	98.5-101.4	>8	N/A	[1]
CE-conductivity detection	1.5	94-108	>6	N/A	[2]
Polypyrrole-doped membrane	65.9	101.5-102.0	<3	N/A	[3]

*Calculated as 20 bands were semultaneously separated and evaluated on a plate.

[1] Bruno SNF, Cardoso CR, Maciel MMA, Vokac L, da Silva Junior AI. Selective identification and quantification of saccharin by liquid chromatography and fluorescence detection. Food Chem 2014;159:309-15.

[2] Bergamo AB, Fracassi da Silva JA, de Jesus DP. Simultaneous determination of aspartame, cyclamate, saccharin and acesulfame-K in soft drinks and tabletop sweetener formulations by capillary electrophoresis with capacitively coupled contactless conductivity detection. Food Chem 2011;124:1714-7.

[3] Álvarez-Romero GA, Lozada-Ascencio SM, Rodríguez-Ávila JA, Galán-Vidal CA, Páez-Hernández ME. Potentiometric quantification of saccharin by using a selective membrane formed by pyrrole electropolymerization. Food Chem 2010;120:1250-4.
